# Systemic Hurdles, Not Cardiologist Awareness: The Barriers to CPET Availability in Japan

**DOI:** 10.1002/joa3.70251

**Published:** 2025-12-11

**Authors:** Takahiro Kamihara, Takuya Omura, Atsuya Shimizu

**Affiliations:** ^1^ Department of Cardiology National Center for Geriatrics and Gerontology Obu Japan; ^2^ Department of Metabolic Research National Center for Geriatrics and Gerontology Obu Japan

**Keywords:** cardiologist, CPET, DPC

To the Editor,

We read with great interest the article “Factors Influencing the Availability of Cardiopulmonary Exercise Testing for Patients Undergoing Cardiac Resynchronization Therapy in Japan,” which appeared in your esteemed journal [1]. The paper provides valuable data by revealing the remarkably low rate of cardiopulmonary exercise testing (CPET) among patients who have undergone cardiac resynchronization therapy (CRT) implantation in Japan. This finding is crucial as it highlights the current status and challenges of CPET dissemination in Japanese healthcare. However, we believe that the interpretation and conclusions of this study overlook some fundamental issues within Japan's medical infrastructure and contain several logical inconsistencies that warrant discussion.

The first point is the logical inconsistency between infrastructure and awareness. The “Discussion” section of the article accurately points out a structural problem: only 21% of medical facilities in Japan are equipped with CPET devices. Despite this, the “Conclusion” proposes that emphasizing the “importance of CPET” is the primary solution to improve its implementation rate. This represents a logical leap, as it ignores the fundamental issue of a lack of necessary equipment and instead reduces the problem to one of physician awareness. The implementation rate cannot be substantially improved without the necessary infrastructure, regardless of how much its importance is emphasized. Similar to the regional disparities observed in catheter ablation, which is more widely adopted than CRT, the lack of infrastructure is a key factor [2]. It is highly probable that in facilities with the equipment, medical professionals are already actively performing CPET for their patients, but their capacity is limited. Conversely, in facilities without the equipment, healthcare providers cannot perform CPET even if they understand its importance. The conclusion of this study risks sending an incorrect message to the medical community—that the low CPET adoption rate is due to a lack of effort from practitioners, rather than a systemic issue. This is a claim made without sufficient scientific evidence.

Furthermore, we note the limits of DPC data and the over‐generalization of conclusions. The authors themselves acknowledge a limitation: the Diagnosis Procedure Combination system (DPC) dataset used in the study only covers a fraction of all hospitals in Japan. Yet, they generalize the conclusion that “the CPET implementation rate is only 3%” to represent the entire country. Many hospitals operating on a fee‐for‐service basis are not included in the DPC dataset [3]. These hospitals have a financial incentive to perform more tests and procedures, and thus may be more actively conducting CPET than their DPC‐participating counterparts. Therefore, the data presented in this study merely reflect the reality within DPC. To extrapolate these findings to the entire nation is a logical contradiction that overlooks the inherent limitations of the data source.

Lastly, there is a discrepancy between CPET safety and implementation rates in older patients. The “Discussion” section cites prior research confirming the safety of CPET in older patients, suggesting that age should not be a barrier to its use. However, the “Results” section shows that the non‐CPET group was significantly older, and the “Conclusion” identifies age over 70 as a factor influencing non‐implementation. This contradiction—that age is a barrier despite its proven safety—is a major weakness of the article's argument. The true reason for this discrepancy may not be a simple matter of medical staff awareness, but rather more nuanced clinical factors not captured by the DPC data, such as frailty [4] or an acute, unstable condition.

In conclusion, we believe that improving the CPET implementation rate requires more than just awareness campaigns. It demands a more fundamental approach that addresses the lack of equipment and human resources in regional medical facilities.

## Funding

The authors have nothing to report.

## Disclosure

No human participant was involved in this study.

## Ethics Statement

The authors have nothing to report.

## Consent

The authors have nothing to report.

## Conflicts of Interest

The authors declare no conflicts of interest.

## Data Availability

In this letter, no data analysis is performed.
